# Injury, Risk and Training Habits Among Dog Agility Handlers: A Cross-Sectional Study

**DOI:** 10.3390/jfmk10030263

**Published:** 2025-07-12

**Authors:** Andrea Demeco, Laura Pinotti, Alessandro de Sire, Nicola Marotta, Antonello Salerno, Teresa Iona, Antonio Frizziero, Dalila Scaturro, Giulia Letizia Mauro, Umile Giuseppe Longo, Antonio Ammendolia, Cosimo Costantino

**Affiliations:** 1 Physical and Rehabilitative Medicine, Department of Medical and Surgical Sciences, University of Catanzaro “Magna Graecia”, 88100 Catanzaro, Italy; iona@unicz.it (T.I.); ammendolia@unicz.it (A.A.); 2 Research Center on Musculoskeletal Health, MusculoSkeletalHealth@UMG, University of Catanzaro “Magna Graecia”, 88100 Catanzaro, Italy; nicola.marotta@unicz.it; 3 Department of Medicine and Surgery, University of Parma, 43126 Parma, Italy; laura.pinotti@unipr.it (L.P.); antonello.salerno@unipr.it (A.S.); cosimo.costantino@unipr.it (C.C.); 4 Physical and Rehabilitative Medicine, Department of Experimental and Clinical Medicine, University of Catanzaro “Magna Graecia”, 88100 Catanzaro, Italy; 5 Department of Biomedical, Surgical and Dental Sciences, University of Milan, 20122 Milano, Italy; antonio.frizziero@unimi.it; 6 Precision Medicine in the Medical, Surgical and Critical Care Areas, University of Palermo, 90100 Palermo, Italy; dalila.scaturro@unipa.it (D.S.); giulia.letiziamauro@unipa.it (G.L.M.); 7 Fondazione Policlinico Universitario Campus Bio-Medico, 00128 Roma, Italy; g.longo@unicampus.it; 8 Research Unit of Orthopedic and Trauma Surgery, Department of Medicine and Surgery, Università Campus Bio-Medico di Roma, 00128 Roma, Italy

**Keywords:** rehabilitation, musculoskeletal diseases, dog agility, sports, injury

## Abstract

**Background**: Dog agility is a rapidly growing sport involving a partnership between a dog and the handler, running through an obstacle course. Despite its increasing popularity and physical benefits, research on handler injuries remains limited. This study aimed to assess injury epidemiology of athletes practicing dog agility. **Methods**: This cross-sectional study was conducted using a comprehensive online survey consisting of 124 items, available in both English and Italian. The questionnaire was divided into four sections: Introduction collected demographic data and medical history; Materials and Methods focused on agility-related activities; Results explored injuries sustained in the past 12 months; Discussion examined training habits unrelated to agility. **Results**: Among 389 participants, the most represented age group ranged between 30 and 40 years old. Overall, 7% reported upper limb injuries, while 27% experienced at least one lower limb injury. Additionally, 20% of participants used medication, and 25% reported at least one chronic illness. On average, handlers trained twice per week and competed in two events per month. Lower limb injuries were predominantly muscular (49%) or ligamentous (14%) and most commonly occurred on grass pitches (56%). These injuries were more common in participants with a higher BMI, those using dynamic handling styles, and those competing at higher levels. **Conclusions**: This cross-sectional study highlighted the importance of identifying risk factors associated with dog agility handlers. Lower limb injuries were the most common, often associated with increased physical demands and handling styles involving intensive running and correlated with reduced physical fitness. Athletic conditioning, including structured warm-up and cool-down practices, might help decline injury risks.

## 1. Introduction

Dog agility is a rapidly growing sport that involves a partnership between a dog and their handler, running together through an obstacle course designed to challenge both their physical and mental skills. This sport is not only a skill test of the dog, but also of the handler’s ability to lead the dog throughout the course. Today, dog agility is probably the most popular dog sport with a 38% increase in participants (870,603 to 1,202,711) between 2009 and 2019 in the United States [1,2]. In 2016, registrations for agility competitions worldwide exceeded 3 million [3]; this growth can be attributed to greater appreciation of the sport, more accessible training fields, and an increase in the number of competitions available. Moreover, physical participation could have positive effects on dog-owners’ and dogs’ health [4].

Dogs of all sizes aged more than 24 months can participate in international competitions. There is no maximum age, because, depending on the breed and size, the physical conditions needed to face a competition might vary greatly. Nonetheless, there are no restrictions on dog breeds; Inkilä et al. [2] reported eighty-eight different breeds practicing the sport, with the most popular ones being Border Collie (16.1%) and Shetland Sheepdog (11.1%) [2].

In agility trials, handlers lead dogs through judge-designed obstacle courses, competing for the fastest and most accurate performance. Scores are assigned to the dog’s performance, with the dog–handler duo advancing through higher levels of competition, based on their achievements. Usually, during a trial day, a dog competes in two to eight runs per day; on the other hand, handlers frequently run multiple dogs, increasing their physical demands [5,6].

The regulations define four categories of dog according to shoulder height (small, medium, intermediate, and large). Furthermore, the actual length of the course ranges between 100 and 200 m, and each “run” lasts on average 30 to 60 s. Depending on the class, a standard set of obstacles must include at least 14 obstacles [7], which the dog must navigate correctly under the guidance of their handler.

Courses are divided into two categories: Agility, including obstacles with contact zones, and Jumping, which includes only jumps and tunnels. In this sport, human athletes are required to perform a variety of movements to effectively guide their dog throughout the course, crucial for providing visual and verbal signals and maintaining the flow of the run. For example, handlers need both to accelerate to keep up with their dog or to get ahead to the next obstacle to provide clear direction and to decelerate quickly, when approaching turns or when they need to slow their dog down for a particular obstacle. Agility courses are also designed with numerous turns and changes in direction so athletes must be agile and have good balance to quickly change direction without losing speed or stumbling [5].

The physical demands of this sport can place handlers at risk for various injuries, such as in other sports, including lower limb muscle strains, ankle sprains, and anterior cruciate ligament injuries due to fast changes of speed and rapid movements [8,9,10,11,12]. Moreover, changes of direction could result in falls or unintended collisions with obstacles or dog, and it should be considered that overuse injuries are frequent considering the repetitive nature of training and competing of this sport [5].

To mitigate these risks, handlers should engage in regular physical conditioning, including strength training, flexibility exercises, and cardiovascular workouts, with proper warm-up and cool-down before and after each training and competition session [13,14,15].

Moreover, Kerr et al. [5] suggested that handlers should invest in appropriate gear, such as supportive footwear that might provide adequate traction, and cushioning impact absorption of running on various surfaces [5]. In addition to personal conditioning, handlers should also be aware of the environmental conditions. Outdoor training and trials can be influenced by the weather; thus, wet or uneven terrain might heighten the risk of slips and falls.

Despite the growing popularity of dog agility and the many changes that have taken place over the past decade, there remains a lack of knowledge regarding the risk of injury to handlers [16].

Most of the existing literature focuses primarily on the dogs. However, two Finnish studies have adopted a more comprehensive approach by analyzing agility-related injuries in competition-level dogs, along with associated risk factors, training methods, and management practices [2,17]. Other studies have focused on specific risk factors or types of injuries in dogs, as well as techniques and approaches to navigating obstacles [1,16,18,19,20]. Only Kerr et al. [5] also analyzed the handlers’ injuries, reporting data on the epidemiological dimensions of injuries affecting both handlers and dogs engaged in agility competitions. However, with only 31 injuries recorded for handlers, much remains unknown about this population.

Thus, the aim of this cross-sectional online survey was to investigate the physical characteristics, athletic performance, training, and injuries in a large sample of dog handlers in order to have a deep insight on injuries and related risk factors in this novel sport.

## 2. Materials and Methods

### 2.1. Survey Development

In this cross-sectional study a four-section online survey using the Office Forms (Microsoft, Redmond, WA, USA) suite, with 124 items in English-Italian language, was administered to a sample of dog agility handlers. The aim of these questions was to gather data on demographic, medical history, agility training methods, non-agility training habits, and histories of injury. The survey was developed in consultation with two experienced agility handlers to ensure a comprehensive evaluation of risk and for accurate terminology. To minimize bias, the survey was anonymous, and a preliminary pilot testing was conducted with five agility handlers to refine question clarity and assess time requested to answer. Adjustments included simplifying technical terms and reordering sections for logical flow. The demographic and non-agility training questions were inspired by another online study administered to female recreational netball players [21]. It was subsequently revised based on feedback and reviewed by physicians for clinical accuracy.

Participants were recruited via social media platforms, adhering to the initiative voluntarily. We created a post, with all the essential information, a brief description of the research objectives, an introduction to the researchers, and a link to the questionnaire to be completed. We targeted both Italian and international athlete groups related to the discipline, after obtaining consent from the group administrators.

Inclusion criteria were dog agility handlers aged 18 years or over, regardless of experience level, and ability to complete the entire online survey. Exclusion criteria were no given consent, and handlers under 18 years of age. Ethical approval was obtained for this study by the REB—Research Ethics Board of the University of Parma, with approval number 55-2023-N on 7 December 2023, ensuring adherence to ethical research practices. The first page included a statement of consent, a privacy agreement, and a link to a document explaining the purpose of the study and the estimated completion time.

### 2.2. Survey Sections

Section 1 gathered demographics and medical information including sex, age, country of residence, height, weight, chronic conditions, medications, and vitamin D supplementation.

Section 2 assessed agility-related activities, including current competition grade, years practicing agility, duration and frequency of weekly sessions, monthly competitions and seminars, primary training goals, warm-up and cool-down routines, handling style, and footwear choices.

Section 3 focused on injuries sustained in the last 12 months, categorized into upper and lower limb injuries. It recorded details such as the number of injuries, the context (training or competition), the timing (early or late in the session), the field surface, diagnosis, injury type, triggering movements or events, and recovery time.

For knee injuries specifically, additional questions addressing training modifications, the need for injections or surgeries, and other related factors were added based on a previous study [21]. Injuries were defined, following prior research, as “any injuries that then stopped you from participating in one or more of the following training sessions of matches” [21,22,23,24].

Section 4 investigated training habits other than agility, including the type, duration, frequency, goals, and perceived benefits of these activities for dog agility handlers [5,21]. The survey consisted of both multiple-choice and open-ended questions to balance analyzable data with the flexibility for participants to provide detailed information.

An example of the survey is described in Supplementary Materials S1.

### 2.3. Statistical Analysis

Survey responses were exported to Microsoft Excel (Microsoft, Redmond, WA, USA), and analyzed using SPSS 30.0.0 (SPSS Inc., Chicago, IL, USA) software. Descriptive statistics were calculated for demographic and agility training data, including means and standard deviations. Variables identified in the content analysis were grouped, and the frequency of responses for each theme was recorded. Absolute (n) and relative (%) frequencies of responses, along with the number of injuries per participant, were calculated. Exposure was estimated by multiplying each athlete’s weekly training frequency by session duration, then extrapolating to the yearly total and summing across all participants.

For competitions, the number of monthly competitions was multiplied by 12, assuming 30 min per event, and summed across the cohort.

Injury incidence × 1000 h was then calculated using:

$$Incidence=1000\times \frac{\sum injuries}{\sum exposure\;hours}$$

Total incidence referred to all injuries and total exposure, while setting-specific incidence was computed separately for training and competition. As a first step, correlations were examined between specific categories (e.g., competition level, running style). Subsequently, univariate linear regressions were conducted to predict the likelihood of injuries among handlers.

## 3. Results

### 3.1. Demographic Data and Medical History

Of 401 handlers who completed the survey, 389 met the inclusion criteria. The majority (57%, n = 222) of respondents were from Italy, followed by participants from other European countries (30%, n = 117), in particular the United Kingdom (3%, n = 12) and Germany (3%, n = 13); other respondents were from North America (5%, n = 20), Australia and New Zealand (5%, n = 20), South America (1%, n = 4), Africa (1%, n = 4), and Asia (1%, n = 2). Most handlers were female (85%). Age, BMI, vitamin D intake, duration of time practicing dog agility, and the number of weekly training sessions and their duration are described in Table 1.

### 3.2. Training Habits

One hundred and eleven handlers (29%) compete in grade 1, 61 (16%) in grade 2, and 215 (55%) in grade 3. Fifty-six (14%) athletes are members of their national team. “Handling style”, defined as the athletic technique used to guide the dog through obstacles, either by closely accompanying it or leading it from a distance, has been classified into three levels: most of the handlers (70%) tend to run with the dog for more than 50% of the course, 78 (20%) run close to the dog for the entire course, and 40 (10%) mostly guide their dog from a distance. Two hundred and twenty-seven (58%) athletes perform a warm-up before each training and each competition, 116 (30%) do it occasionally, and 46 (12%) never do it; this variable has been found to be related to the reported injuries. Only 159 (41%) handlers follow a cool-down routine after training/competition, 120 (30%) do it occasionally, and 110 (29%) never do it. The most used shoes are trail running shoes (81%), followed by running shoes (10%), light shoes (7%), soccer shoes (3%), and normal sneakers (1%). The main reason handlers train was recorded as a multiple-choice response: to engage in activities with their dog (n = 353), followed by improving agility skills (n = 309), enhancing performance (n = 280), socializing (n = 93), socializing their dog (n = 81), fitness training (n = 61), and weight loss (n = 16).

### 3.3. Non-Agility Training Habits

Among surveyed handlers, 256 (66%) participated in at least one other sport. Of these 35% trained twice weekly, 31% once weekly, and 34% for three or more sessions per week (mean 1.41 ± 1.34 per week). Session durations were typically 60 min (62%) with an average duration of 68 ± 44 min. In Table 2, activities and the number of handlers doing them are explained (multiple choice question).

### 3.4. Injuries

In the last 12 months prior to completing the survey, 30 upper limb injuries were reported by 26 different handlers (7% of the total responding). Of these, 22 handlers experienced only one injury (85%). Most of these injuries occurred during training (61%), specifically during the second half of the training session (64%). Only 9% of the injuries occurred on sand, 13% on synthetic fields, and 78% on grass. Among diagnosed injuries, 66% were muscular injuries, 26% were bone fractures, and 8% were ligament tears. The primary causes of these injuries included change of direction (35%), collision with the dog (22%), falls (17%), collision with field obstacles (17%), and other causes (17%). Other characteristics such as surface, cause of injury, and type of injury are also described in Table 3.

In the same period 105 handlers (27%) reported at least one lower limb injury, 26 (7%) experienced two injuries, and three handlers (1%) reported three injuries for a total of 137 lower limb injuries. Focusing on knee injuries, these accounted for 39 cases (37% of total lower limb injuries). Most of them (63%) occurred during training, distributed almost equally in the first and the second halves of it. The surfaces most involved in lower limb injuries were grass (56%), synthetic fields (34%), and sand (10%). Common diagnoses included muscular lesion (56%), ligament tears 24%, cartilaginous lesions 6%, combined ligament and cartilaginous lesions (8%), and bone fractures (6%). The causes of knee injuries were predominantly changes in direction (76%), collisions with dogs (7%), collisions with obstacles (2%), and falls (15%). Rest periods following injuries varied: 11% required no rest, 7% less than 10 days, 41% between 10 days and one month, 32% between one and three months, and 9% more than three months. Note that in 100% of second injuries (regardless of the type of injury) the rest time was greater than one month (but less than 3).

A higher BMI is related to the male population (Pearson’s r = 0.127, *p*-value = 0.012), shorter training time (Pearson’s r = 0.124, *p*-value = 0.014), and a more static handling style (Pearson’s r = 0.138, *p*-value = 0.007). Taking vitamin D is associated with a lower percentage of lower limb injuries (Pearson’s r = 0.123, *p*-value = 0.015) and female sex (Pearson’s r = 0.129, *p*-value = 0.011). Athletes who train more times on a weekly basis (Pearson’s r = 0.186, *p*-value < 0.001) and compete more times a month (Pearson’s r = 0.295, *p*-value < 0.001) tend to reach a higher performance level; these athletes perform a warm-up (Pearson’s r = 0.198, *p*-value < 0.001) and cool-down (Pearson’s r = 0.212, *p*-value < 0.001) routine more often and also participate in sports other than just dog agility (Pearson’s r = 0.1, *p*-value = 0.05). Athletes who have been practicing dog agility for longer periods tend to train several times a week (Pearson’s r = 0.137, *p*-value = 0.007) and consistently perform a warm-up routine (Pearson’s r = 0.157, *p*-value = 0.002); they also usually adopt a more static handling style (Pearson’s r = 0.198, *p*-value < 0.001). A shorter agility training session is associated with a higher number of lower limb injuries (Pearson’s r = 0.136, *p*-value = 0.007).

Engaging in three to five agility training sessions per week significantly increases the likelihood of reaching a higher competitive level (*p* = 0.005, 0.038, 0.043; t = 2.837, 2.086, 2.032).

Engaging in an additional sports activity three times a week improves competitive performance (*p* = 0.019, t = 2.361). Male athletes are more likely to use a handling style focused on running (*p* = 0.016, t = 2.421). Regarding injury risk, younger athletes (18–30 years old) are less likely to experience upper limb injuries (*p* = 0.043, t = 2.032), while vitamin D supplementation appears protective against lower limb injuries (*p* = 0.015, t = 2.436).

Nevertheless, higher levels of competition are not without risks. Participating in six or more competitions a month tends to be associated with a higher risk of lower limb injuries (*p*-value 0.038, t-value 2.078), while training for 60 min or more per session has a protective effect against lower limb injuries (*p*-value 0.05, t-value 1.960). Handlers who have been practicing agility for several years face an increased risk of knee injuries (*p*-value < 0.001, t-value 3.992).

## 4. Discussion

The aim of this study was to examine and describe the population of dog agility handlers to better understand their demographic factors, physical workout, and the incidence of injuries.

Athletes from all over the world responded to this survey, showing a strong interest and awareness of dog handlers to obtain an insight into the sport’s demands and their risk of injury. There was a clear predominance of Europeans, especially Italians; this geographic characteristic can be attributed to the survey’s dissemination via social media platforms by Italian researchers; however, the bilingual nature of the questionnaire (English/Italian) allowed for contributions from other countries, including a significant input from the United States where the sport is particularly widespread and growing [1]. Confirming the results of previous studies, there is a high female participation in this sport, with women being over 80% [5,25]. This demographic detail may explain the higher number of athletes taking vitamin D, as women in both the general population [26] and among athletes [27,28] tend to be more deficient in vitamin D than men. A meta-analysis of 2300 athletes has shown that 56% had inadequate levels of vitamin D, and there is also a correlation with the type of activity performed, with higher deficiency rates among indoor athletes due to reduced exposure to UV rays [29]. The growing interest in the optimal range of vitamin D is due to its strong correlation with better muscular performance, explosive strength, running speed, lactate threshold, and aerobic power [30,31]. Despite this, there is still no consensus on the type of supplementation in terms of type (cholecalciferol or calcifediol), frequency (daily/weekly/monthly), and dosages [32,33].

Although there are only a few descriptive studies, our data agrees with those found in a recent study [5] regarding age and BMI distributions. However, our cohort was younger but more experienced than that described by Cullen et al.’s [25] study: this is likely due to the increased popularity of dog agility over the past 10 years and the rise in competitive levels and competitions, making it more appealing even to younger athletes. Furthermore, our study demonstrates that individuals with a higher BMI tend to engage in lower levels of physical activity; this may lead to a more static handling style, which requires less physical effort.

The handling style, defined as the athletic technique used to guide the dog through obstacles, reported that most athletes use a combination of handling techniques that involve sending the dog independently over some obstacles and guiding it closer through the more challenging parts of the course, providing the dog with more visual and vocal cues. This aspect is particularly crucial for identifying the type of training necessary to enhance the performance of athletes. Indeed, the appropriate training regimen, such as high-intensity interval training (HIIT), can significantly improve the most utilized handling techniques, providing athletes with the competence, skills, and strength required to better perform at an advanced level, being able to choose the correct distance from the dog to guide it. As reported in various studies [34,35,36] focusing on sports where accelerations and decelerations are critical for performance, the use of HIIT optimizes cardiovascular efficiency, muscular strength, and anaerobic performance in athletes, thereby highlighting its positive impact on metabolic adaptation [37]. Furthermore, a combined handling style may represent a protective factor against injuries resulting from changes of direction, collisions with the dog, or collisions with the field obstacles. A dynamic handling style, characterized by running close to the dog for the entire course or for more than 50% of it, could increase the risk of such injuries; conversely, a more static approach, involving reduced movement and greater spatial control, may help to reduce the risk of collisions with both the dog and obstacles.

Of the total of 167 injuries reported among agility dog handlers, in 160 the information about time lost from activity was available, allowing classification by severity. Among these, 51 injuries (32%) were considered mild, involving less than 7 days of absence; 27 (17%) were moderate, lasting between 8 and 28 days; and 82 (51%) were classified as severe, resulting in more than 28 days away from activity. On average, handlers lost around 34 days for each injury, underlining a considerable disruption to their regular training routines and potentially affecting the continuity of their work with their dogs.

In terms of exposure, handlers accumulated a total of 35,963 h, including 31,655 h of training and 4308 h of competition (each competition was classified as half an hour of activity). When all injuries were taken into consideration, the overall incidence was 4.64 injuries per 1000 h of exposure; however, dividing the injuries by setting, a clear difference emerged: during training, the incidence was 2.75 injuries per 1000 h, whereas during competition it climbed to 11.84 injuries per 1000 h. This pattern, a considerably higher injury risk in competitive settings, is consistent with what has been observed in other sports. For example, in football, handball, and basketball, training injury rates generally range from two to five per 1000 h, but rates during matches can be well beyond 20 injuries per 1000 h due to increased intensity and unpredictable dynamics [38,39,40,41,42]. Even in sports with a lower degree of physical contact, such as volleyball or athletics, competitions are recognized as riskier moments compared to training [43,44,45,46].

In the context of dog agility, this pronounced gap between training and competition injury rates likely reflects the greater physical and mental demands placed on handlers during competitions, along with factors like increased stress, faster movements, and the more dynamic interactions required over the course, and suggests that injury-prevention programs should pay special attention to supporting handlers during competitive events, where their risk appears to spike.

Equally notable is the high proportion of severe injuries, with more than half of the cases involving over 28 days away from activity. Such a significant burden does not just affect the handlers’ athletic progress, but also has the potential disturb to the delicate balance of teamwork with their dogs. This underscores the critical role of comprehensive athletic preparation in dog agility, as already widespread for other athletic disciplines [47]. A well-structured training regimen, complemented by the consistent implementation of warm-up and cool-down routines, could indeed significantly mitigate the risk of those injuries [48], reducing the incidence of injuries associated with changes of direction, identified in this study as a frequent cause of harm [49].

Another important aspect concerns the risk of relapse or second injury, which, according to other studies [50], results in longer recovery times and thus extended periods away from competition; in this context, a specialized training program focused on recovery and the prevention of re-injury is essential.

In particular, injuries occurred more frequently during training then competitions in agility, aligning with findings from other sports as soccer [50]; this could be due the structure of training sessions, often with prolonged running phases and exercises. In fact, a 45-min training session in dog agility corresponds on average to four or five complete runs of the entire course, often with the addition of exercises aimed at overcoming specific sequences; by comparison, an average competition corresponds to only two runs of two different courses, for a total of about 2 min of running and sprinting.

The lower limb injury patterns seen among dog agility handlers were also comparable to those reported in other high-intensity sports that involve sudden direction changes and explosive movements, such as basketball [39], soccer [51], and tennis [52]. In these sports muscular tears are also the most common injuries recorded due to the repetitive movement. Likewise, agility handlers experienced persistent biomechanical stressors, especially during rapid acceleration and deceleration to maintain the pace of their dogs. Comparisons with soccer are particularly interesting, because these two sports are played on grassy surfaces where uneven ground, moisture, and mud all play a major role in falls or slips. In fact increased prevalence of lower limb injuries in dog agility handlers is consistent with trends of overuse and acute injuries of the muscles, ligaments, and joints commonly seen in soccer players [39]. In particular, in football, lower limb injuries account for 60–90% of all injuries, with muscle/tendon injuries occurring at an incidence rate of 6.8 per 1000 h of exposure. Moreover, injury incidence in both sports shows a direct association with the number of hours spent in competition [53,54]. Additionally, our results show that the incidence of lower limb injuries is higher in individuals who engage in fewer training sessions. This may be attributed to a reduced athletic capacity and insufficient development of the required technical gestures, which predisposes individuals to various types of injuries resulting from the frequent directional changes involved in the dog agility discipline.

Upper limb injuries were understandably less common in a non-contact sport where athletes primarily use their arms for balance [55]. Nevertheless, these injuries have been studied to assess whether falls and collisions pose a significant risk for handlers. In our study, falls accounted for 17% and collisions constituted 31% of the total upper limb injuries. Notably, due to the more traumatic nature of these events, the upper limb exhibited a higher incidence of fractures then the lower limb, as also documented by Andersson et al. [56], and confirmed in our findings (26% of fractures in the upper limb compared to 6% in the lower limb).

Moreover, the findings of this survey highlighted that engaging in sports activities other than dog agility training provides significant benefits, particularly in terms of athletic performance, improving strength, balance, and flexibility, meeting the biomechanical demands of dog agility. Athletes who incorporate complementary disciplines into their training regimens demonstrated an improved capacity to sustain high-intensity activities while reducing the risk of sudden movements or imbalances. It is interesting to note that people who practice sports other than dog agility are more likely to implement warm-up and cool-down protocols. This suggests that practicing other sports may influence the execution of static and dynamic warm-up and cool-down exercises, and this, as our data suggests, could be an additional factor that improves performance in dog agility.

Thus, current training practices might not address sufficiently the specific sport demands, showing the need for tailored athletic preparation protocols, incorporating strength, balance, and flexibility training, besides well-planned warm-up and cool-down routines, which could help reduce injury risks [57,58,59,60,61,62]. Such programs should help handlers cope with the high pace and dynamic nature of their sport, reducing the likelihood of sudden movements and disparities between surfaces [63,64].

Moreover, warm-up is more widespread than cool-down, with 88% of athletes doing it at least a few times before training and competitions. Only a smaller number of athletes practice a cool-down routine, which may be a missed chance to reduce the risk of exercise-induced stiffness and subsequent injuries [65,66]. This is consistent with the results of at least one other study carried out on runners, where the control group carried out significantly more warm-up (20.8%) than cool-down (8.3%) [63,67,68,69].

Although not statistically significant, the data on the surface where injury occurred provides further insight: in fact grass, the most commonly used surface for agility fields, carries risk factors such as moisture, uneven sections, and mud (as already found in other sports [52]), all of which contribute to a higher likelihood of falls and collisions.

Given the high physical demands and risk factors discussed, the choice of footwear becomes a critical consideration to enhance performance and prevent injuries. The major use of trail running shoes (81%) might be justified and considered consistent with the most frequently reported surface: grass. This type of footwear offers a good balance between stability during running and responsiveness during changes of direction and it is also more structured than a typical sneaker or cleat [70]. Handlers’ trail running shoes might be an indication of the need for suitable equipment, but future studies are warranted to evaluate its effect in reducing the potential risk associated with changes in direction and variability in running surface.

This study was not without limitations. Firstly, the use of an online survey as the primary data collection tool introduced potential biases, such as self-selection, where only highly motivated or aware participants might have responded; furthermore, the self-reported nature of the injuries posed risks of inaccuracy, particularly regarding details such as diagnosis and injury severity. Additionally, the sample selection was inherently influenced by the distribution of the survey within social media groups predominantly composed of Italian participants, leading to a significant geographical bias. The sample displayed a marked gender imbalance, with a substantial predominance of female respondents (85%), which may limit the generalizability of the findings to male handlers. Moreover, while the survey aimed to cover a comprehensive range of injuries by allowing respondents to detail and differentiate between multiple incidents, this approach led to challenges in data analysis: specific details such as the precise location of injuries and the diagnostic accuracy of self-reported cases were often difficult to ascertain. A future field-based study could address these limitations by ensuring a more diverse and balanced sample in terms of geography and gender, to enable a more precise categorization of injuries and provide the foundation for focused research on smaller but more representative cohorts. Nevertheless, the ability to rapidly gather data from a large population in this study significantly enhanced the statistical power of the findings and represents a considerable achievement in the field.

## 5. Conclusions

In conclusion, this study laid the groundwork for advancing the safety, performance, and health of agility dog handlers, fostering collaboration between athletic trainers, physiotherapists, and agility organizations to address the unique challenges of this growing sport. There is a relatively high incidence of lower limb injuries, mostly muscular and ligamentous. Our results highlight the need of a structured training protocol to adequately target the sport-related biomechanical demands, including rapid direction changes, explosive accelerations, and balance on uneven terrain.

## Figures and Tables

**Table 1:** Descriptive data of dog agility handlers (sample n = 389).

Age (y)	29.7 ± 5.1
Female	85%
BMI (kg^2^/mt)	24.4 ± 6.3
Vitamin D supplementation (% of participants)	31%
Duration of time practicing dog agility (y)	11.7 ± 7.0
Weekly training session (n)	2.1 ± 0.8
Length of training session (min)	45 ± 22.5

**Table 2:** Participation in activities other than dog agility.

Activity	Number of Handlers Doing It
Weight training—resistance machines	38
Weight training—free weight (e.g., dumb bells, bar bells)	77
Plyometric training—jumping/hopping/bounding	55
Balance training	51
Class/group-based training—weight bearing (circuit training, body pump)	27
Class/group-based training—non-weight bearing (e.g., spinning)	11
Body weight training—calisthenics (e.g., press up, pull ups)	52
Yoga	55
Cross-fit	13
Distance running	76
Distance cycling	18
Dog obedience	34
Disc dog	5
Canicross, Bikejoring	26
Other	103

**Table 3:** Injury characteristics. Mild, <7 days out of sport; moderate, between 7 and 28 days out of sport; severe, >7 days out of sport.

Injury Characteristics		Upper Limb	Lower Limb
Surface	Grass	78%	56%
	Synthetic field	13%	34%
	Sand	9%	10%
Type of injury	Muscle injury	66%	56%
	Bone fracture	26%	6%
	Ligament tear	8%	24%
	Cartilaginous lesion		6%
	Ligament + Cartilaginous lesion		8%
Cause	Change of direction	35%	76%
	Collision with dog	22%	7%
	Collision with obstacle	22%	2%
	Fall	17%	15%
	Other	17%	
Injury severity	Mild	32%	
	Moderate	17%	
	Severe	51%	
Exposure (h)	Training	31,655	
	Competition	4308	
	Total	35,963	
Injury setting if reported (n)	Training	87	
	Competition	51	
Incidence (injuries per 1000 h)	Training	2.75	
	Competition	11.84	
	Total	4.64	

## Data Availability

Please add the corresponding content of this part.

## Supplementary Materials

The following supporting information can be downloaded at: https://www.mdpi.com/article/10.3390/jfmk10030263/s1, Survey.
