# Supplementary material for: Injury, Risk and Training Habits Among Dog Agility Handlers: A Cross-Sectional Study
Source: J Funct Morphol Kinesiol. 2025 Jul 12;10(3):263. doi: 10.3390/jfmk10030263 (PMC12286276; doi:10.3390/jfmk10030263)
Supplement: Supplementary file 1 [file jfmk-10-00263-s001.zip › jfmk-3691463-supplementary.pdf]

Injury in Dog Agility Athletes

If you have 10 min to answer to this survey thank you! Se hai 10 min per rispondere al questionario grazie!
Who am i? Why i care? Click the link to find out! Chi sono io? Perchè mi interessa? Segui il link per scoprirlo!
https://docs.google.com/document/d/1gIgYgQ0taPLo445cVU1RlJxpR09RPChdKuU9firhAWY/edit?usp=drive_link

* Obbligatoria

Informativa privacy - Consenso Informato

https://docs.google.com/document/d/128Uyx1UVPhQECxn3FjiuTuOwuTUeIz4uUvAXUEGGzG4/edit?usp=drive_link

https://drive.google.com/file/d/1HTtdlit6RtmG_7N8ATXQbgTNurx-wGeU/view?usp=drive_link

1. I, (first name and last name) *

   Il sottoscritto/a (nome e cognome)

2. Live in (country, city, street) *

   Residente in (paese, città, via)

3. Declares: *

Dichiara:

- [ ] to have read the recived informative form, to have understood the information in the form and to have had the time and opportunity to ask questions and have satisfying answers from the staff member. *-di aver letto il suddetto foglio informativo ricevuto, di aver compreso le informazioni in esso contenute e di aver avuto ampio tempo ed opportunità di porre domande ed ot tenere risposte soddisfacenti dal personale addetto;-*
- [ ] to have understood that the participation in the study is entirely free and voluntary, that you may at any time terminate participation in the study without having to provide any reason. *-di aver compreso che la partecipazione allo studio è del tutto volontaria e libera, che ci si potrà ritirare dallo studio in qualsiasi momento, senza dover dare spiegazioni e senza che ciò comporti alcuno svantaggio o pregiudizio;-*
- [ ] to have understood the nature and activities of the study and the related risks; *-di aver compreso la natura e le attività che la partecipazione allo studio comportano e i relativi eventuali rischi;-*
- [ ] to have understood that participation in this study will not lead to the recognition of any direct or indirect economic advantage. *-di aver compreso che la partecipazione a questo studio non comporterà il riconoscimento di alcun vantaggio di natura economica diretto o indiretto.-*

4. Consequently, the undersigned consent/does not consent to participate in the study, in the knowledge that such consent is freely expressed and is revocable at any time without this entailing any disadvantage or prejudice. *

Conseguentemente, il/la sottoscritto/a acconsente/non acconsente a partecipare allo studio, nella consapevolezza che tale consenso è manifestato liberamente ed è revocabile in ogni momento senza che ciò comporti alcuno svantaggio o pregiudizio.

- ◯ CONSENT/*ACCONSENTO*
- ◯ DOES NOT CONSENT/*NON ACCONSENTO*

5. Are you over 18? *

Sei maggiorenne?

- ◯ Yes
- ◯ No

6. The questionnaire does not collect personal data that directly or even potentially identify those concerned. For the collection of responses, the Microsoft Form platform is used, an app owned by Microsoft Corporation which could collect additional user data (e.g. IP address, location, etc.) in accordance with its terms of service and its own privacy legislation that you accept by using the service and can be consulted at this link https://privacy.microsoft.com/it-it/privacystatement

Il questionario non raccoglie dati personali che identificano in maniera diretta o anche solo potenziale i diretti interessati. Per la raccolta delle risposte viene utilizzata la piattaforma Microsoft Form, app di proprietà di Microsoft Corporation che potrebbe raccogliere ulteriori dati degli utenti (es. indirizzo IP, localizzazione, etc.) in conformità ai propri termini di servizio e la propria normativa privacy che accetti utilizzando il servizio ed è consultabile a questo link https://privacy.microsoft.com/it-it/privacystatement

- ◯ Understood / capito

Who are you?

Parlaci di te!

The survey avarage time is 5 to 10 min, depending on how many injuries you had. Please stay at least for section 2 (this one), 3 and 4. If 5 is too long for you you can go to the end and send the survay. Thank you!

Il tempo di compilazione medio è di 5/10 minuti che dipende dal numero di infortuni avuti. Ti prego di compilare le sezioni 2 (questa) 3 e 4, in quanto più importanti. Se la 5 è troppo lunga puoi andare alla fine ed inviare il questionario. Grazie!

7. How old are you? *

Quanti anni hai?

- ◯ 18 - 25
- ◯ 25 - 30
- ◯ 30 - 35
- ◯ 35 - 40
- ◯ 40 - 45
- ◯ 45 - 50
- ◯ 50 - 60
- ◯ > 60

8. What's your sex?

Qual'è il tuo genere?

- ◯ Woman - *Donna*
- ◯ Man - *Uomo*
- ◯ Prefer not to say - *Preferisco non rispondere*

9. Which country do you live and do agility dog in? *

In quale paese vivi e pratichi agility?

[ ]

10. If you live in Italy, in which region?

Se vivi in iIalia, in quale regione?

- ◯ Valle D'Aosta
- ◯ Piemonte
- ◯ Lombardia
- ◯ Trentino Alto Adige
- ◯ Friuli Venezia Giulia
- ◯ Veneto
- ◯ Emilia Romagna
- ◯ Liguria
- ◯ Toscana
- ◯ Marche
- ◯ Abruzzo
- ◯ Umbria
- ◯ Lazio
- ◯ Calabria
- ◯ Molise
- ◯ Campania
- ◯ Basilicata
- ◯ Puglia
- ◯ Sicilia
- ◯ Sardegna

11. How tall are you? (cm) *

Quanto sei alto/a? (cm)

12. How much do you weight? (kg) *

Quanto pesi? (kg)

13. Do you have any cronical condition? *

Hai patologie croniche?

- ◯ Yes
- ◯ No

14. Do you have any cronical condition, which kind? *

Se hai patologie croniche, di che tipo?

- ☐ yes, cardiological - *cardiologiche*
- ☐ yes, pneumological - *pneumologiche*
- ☐ yes, reumatological - *reumatologiche*
- ☐ yes, neurological - *neurologiche*
- ☐ yes, osteomuscolar - *osteomuscolari*
- ☐ other - *altri*

15. Which one?

Quali?

16. Do you take any drugs (medicine)?

Assumi medicine?

- ◯ Yes
- ◯ No

17. If Yes write them down

Se si specifica quali

18. Do you take any vitamin D supplements? *

Assumi integratori per la vitamida D (dibase)?

- ◯ Yes
- ◯ No

Agility

19. How long have you being doing agility dog? (years) *

Da quanto tempo pratichi agility dog? (anni)

20. How long have you been doing agility at your current level (with your "best" dog)? (years) *

Da quanto tempo pratichi agility al livello attuale (con il tuo cane "migliore")? (anni)

21. What level of agility do you currently play? (plase check all the apply) *

In quale livello di agility competi al momento? (è possibile selezionare anche più di un'opzione)

- ☐ Avviamento
- ☐ Agility 1 (1° brevetto)
- ☐ Agility 2 (2° brevetto)
- ☐ Agility 3 (3° brevetto)
- ☐ National champion - campione nazionale
- ☐ Member of any national team - membro di una nazionale

22. How many session of agility do you usually do? *

Quante sessioni di agility fai?

	1 session	2 session	3 session	4 session	5 session	6 session or more	None, less then 1 a month
Training (weekly) *Allenamento (a settimana)*	○	○	○	○	○	○	○
Competition (monthly) *Gara (al mese)*	○	○	○	○	○	○	○
Seminar (stage) (monthly) (al mese)	○	○	○	○	○	○	○

23. How long does each agility session usually last? *

Quanto dura ciascuna sessione?

	30 min	45 min	1 hour	2 hours	don't do them
Training - *allenamento*	◯	◯	◯	◯	◯
Seminar - *stage*	◯	◯	◯	◯	◯

24. What is your main reason for agility training? (plase check all the apply) *

Qual'è lo scopo principale per cui ti alleni in agility? (seleziona tutte le vere)

- ☐ Improve performance - *migliorare le performance*
- ☐ Improve skills - *migliorare la tecnica*
- ☐ Lose weight - *perdere peso*
- ☐ Improve muscolar tone - *migliorare il tono muscolare*
- ☐ Fitness
- ☐ Socialising - *socializzare*
- ☐ Activity with your dog - *fare attività col cane*
- ☐ Socializing your dog - *socializzare il tuo cane*

25. Do you do any warm up? *

Fai riscaldamento?

- ◯ Yes
- ◯ No
- ◯ Sometimes - *a volte*

26. Do you do any cool down? *

Fai defaticamento?

- ◯ Yes
- ◯ No
- ◯ Sometimes - *a volte*

27. What's your "run" style? *

Qual'è il tuo stile di conduzione?

- ◯ I always run next to my dog - *corro insieme al cane per tutto il percorso*
- ◯ I ty to run next to my dog but in some cases i'll send him doing obstacles by himself - *cerco di correre insieme al cane ma a volte lo invio in autonomia su alcuni ostacoli*
- ◯ I send my dog doing obstacles by himself the majority of the time - *invio il cane in autonomia sugli ostacoli per la maggior parte del percorso*

28. What kind of shoes do you use during training/competition?

Che tipo di scarpe utilizzi durante allenamento/competizioni?

- ◯ Normal Sneaker - n*ormali scarpe da ginnastica*
- ◯ Running shoes - *scarpe da corsa*
- ◯ Trail running shoes (e.g. salomon speedcross, adidas terrex etc)
- ◯ Very light shoes - *scarpe sportive molto leggere*
- ◯ Soccer shoes - *scarpe da calcio (con tacchetti)*

Injury information - *Informazioni sugli infortuni*

For the following questions, an "injury" is defined as "any injury that then stopped you from participating in one or more of the following training session or competition"
Per le seguenti domande, un "infortunio" è definito come "qualsiasi infortunio che ti ha impedito di partecipare ad uno o più allenamenti o competizioni".

29. How many injuries have you had in your upper limb in the last 12 months? *
Quanti infortuni hai avuto agli arti superiori negli ultimi 12 mesi?

- ◯ 0
- ◯ 1
- ◯ 2
- ◯ more

30. Did you injury yourself during training or competition?
Ti sei infortunato/a durante un allenamento o una competizione?

- ◯ training - *allenamento*
- ◯ competition - *competizione*

31. If training in which half of the training did the injury occur?
Se durante un allenamento, durante quale metà dell'allenamento?

- ◯ first half - *prima metà*
- ◯ second half - *seconda metà*

32. If competition, which run did the injury occur?
Se durante una competizione, durante quale "giro"?

- ◯ 1 run
- ◯ 2 run
- ◯ 3 run
- ◯ 4 run
- ◯ any other run

33. On which kind of surface where you running?
Su quale superfice stavi correndo?

- ◯ Grass - *erba*
- ◯ Synthetic grass field - *campo in erba sintetica*
- ◯ Sand - *sabbia*

34. Was the injury diagnosed by someone?
L'infortunio è stato valutato/diagnosticato da qualcuno?

- ◯ Yes
- ◯ No

35. How was your injury diagnosed?
Come è stato diagnosticato il tuo infortunio?

- ◯ Physiotherapist/sport therapist/sport rehabilitator - d*a un fisioterapista/terapista dello sport/riabilitatore sportivo*
- ◯ Doctor - *da un medico*
- ◯ Surgeon - *da un chirurgo*
- ◯ X-ray - *tramite rx (lastra)*
- ◯ MRI - *tramite risonanza magnetica*
- ◯ Other - *altro*

36. In other please describe
Se altro per favore descrivi

[ ]

37. If your injury was diagnosed by someone, what type of injury did you sustain?
Se l'infortunio è stato diagnosticato da qualcuno, quale tipo di infortunio è stato refertato?

- ◯ bone fracture - *rottura ossea*
- ◯ muscle injury - *lesione muscolare*
- ◯ ligament tear - l*esione legamento*
- ◯ cartilage tear - *lesione cartilaginea*
- ◯ ligament + cartilage tear - *lesione legamentosa + lesione cartilaginea*
- ◯ other - *altro*

38. In other please describe

Se altro per favore descrivi

39. How the injury occur?

Come è successo l'infortunio?

- ◯ dog collision - *collisione con il cane*
- ◯ object collision - *collisione con un oggetto*
- ◯ trip - *caduta*
- ◯ changing direction - *cambiamento di direzione*
- ◯ other - *altro*

40. If other, how?

Se altro, come?

41. How long were you unable to do agility training for the result of your injury? (months)

Per quanto tempo sei rimasto a riposo a seguito dell'infortunio? (mesi)

42. Did you injury yourself during training or competition? (other injury)

Ti sei infortunato durante un allenamento o una competizione? (altro infortunio)

- ◯ training - *allenamento*
- ◯ competition - *competizione*

43. If training which half of th training did the injury occur?

Se durante l'allenamento, in quale metà dell'allenamento?

- ◯ first half - *prima metà*
- ◯ second half - *seconda metà*

44. If competition, which run did the injury occur?
Se durante una competizione, durante quale giro?

- 1 run
- 2 run
- 3 run
- 4 run
- any other run

45. On which kind of surface where you running?
Su quale superfice stavi correndo?

- Grass - *erba*
- Synthetic grass field - *campo in erba sintetica*
- Sand - *sabbia*

46. Was the injury diagnosed by someone?
L'infortunio è stato valutato/diagnosticato da qualcuno?

- Yes
- No

47. How was your injury diagnosed?
Come è stato diagnosticato il tuo infortunio?

- Physiotherapist/sport therapist/sport rehabilitator - *da un fisioterapista/terapista dello sport/riabilitatore sportivo*
- Doctor - *da un medico*
- Surgeon - *da un chirurgo*
- X-ray - *tramite rx (lastra)*
- MRI - *tramite risonanza magnetica*
- Other - *altro*

48. In other please describe
Se altro per favore descrivi

49. If your injury was diagnosed by someone, what type of injury did you sustain?

Se l'infortunio è stato diagnosticato da qualcuno, quale tipo di infortunio è stato refertato?

- ◯ bone fracture - *rottura ossea*
- ◯ muscle injury - *lesione muscolare*
- ◯ ligament tear - *lesione legamento*
- ◯ cartilage tear - *lesione cartilaginea*
- ◯ ligament + cartilage tear - l*esione legamentosa + lesione cartilaginea*
- ◯ other - *altro*

50. In other please describe

Se altro per favore descrivi

51. How the injury occur?

Come è successo l'infortunio?

- ◯ dog collision - collisione con il cane
- ◯ object collision - collisione con un oggetto
- ◯ trip - caduta
- ◯ changing direction - cambiamento di direzione
- ◯ other - altro

52. In other please describe

Se altro per favore descrivi

53. How long were you unable to do agility training for the result of your injury? (months)

Per quanto tempo sei rimasto a riposo a seguito dell'infortunio? (mesi)

54. How many injuries have you had in your lower limb in the last 12 months? *

Quanti infortuni hai avuto agli arti inferiori negli ultimi 12 mesi?

- ◯ 0
- ◯ 1
- ◯ 2
- ◯ 3
- ◯ 4
- ◯ 5 or more

55. Did you injury yourself during training or competition? (compleate one of each session for every injury)

Ti sei infortunato durante un allenamento o una competizione? (completa una di queste sezioni per ogni infortunio)

- ◯ training - allenamento
- ◯ competition - competizione

56. If training which half of th training did the injury occur?

Se durante un allenamento, durante quale metà dell'allenamento?

- ◯ first half - prima metà
- ◯ second half - seconda metà

57. If competition, which run did the injury occur?

Se durante una competizione, durante quale giro?

- ◯ 1 run
- ◯ 2 run
- ◯ 3 run
- ◯ 4 run
- ◯ any other run

58. On which kind of surface where you running?

Su quale superfice stavi correndo?

- ◯ Grass - erba
- ◯ Synthetic grass field - campo in erba sintetica
- ◯ Sand - sabbia

59. Was the injury diagnosed by someone?

L'infortunio è stato valutato/diagnosticato da qualcuno?

- ◯ Yes
- ◯ No

60. How was your injury diagnosed?

Come è stato diagnosticato il tuo infortunio?

- ◯ Physiotherapist/sport therapist/sport rehabilitator - da un fisioterapista/terapista dello sport/riabilitatore sportivo
- ◯ Doctor - da un medico
- ◯ Surgeon - da un chirurgo
- ◯ X-ray - tramite rx (lastra)
- ◯ MRI - tramite risonanza magnetica
- ◯ Other - altro

61. In other please describe

Se altro per favore descrivi

62. If your injury was diagnosed by someone, what type of injury did you sustain?

Se l'infortunio è stato diagnosticato da qualcuno, quale tipo di infortunio è stato refertato?

- ◯ bone fracture - rottura ossea
- ◯ muscle injury - lesione muscolare
- ◯ ligament tear - lesione legamento
- ◯ cartilage tear - lesione cartilaginea
- ◯ ligament + cartilage tear - lesione legamentosa + lesione cartilaginea
- ◯ other - altro

63. In other please describe

Se altro per favore descrivi

64. How the injury occur?

Come è successo l'infortunio?

- dog collision - collisione con il cane
- object collision - collisione con un oggetto
- trip - caduta
- changing direction - cambiamento di direzione
- other - altro

65. In other please describe

Se altro per favore descrivi

66. How long were you unable to do agility training for the result of your injury? (months)

Per quanto tempo sei rimasto a riposo a seguito dell'infortunio? (mesi)

67. Did you injury yourself during training or competition? (compleate one of each session for every injury)

Ti sei infortunato durante un allenamento o una competizione? (completa una di queste sezioni per ogni infortunio)

- training - allenamento
- competition - competizione

68. If training which half of th training did the injury occur?

Se durante un allenamento, durante quale metà dell'allenamento?

- first half - prima metà
- second half - seconda metà

69. If competition, which run did the injury occur?

Se durante una competizione, durante quale giro?

- 1 run
- 2 run
- 3 run
- 4 run
- any other run

70. On which kind of surface where you running?
Su quale superfice stavi correndo?

- ◯ Grass - erba
- ◯ Synthetic grass field - campo in erba sintetica
- ◯ Sand - sabbia

71. Was the injury diagnosed by someone?
L'infortunio è stato valutato/diagnosticato da qualcuno?

- ◯ Yes
- ◯ No

72. How was your injury diagnosed?
Come è stato diagnosticato il tuo infortunio?

- ◯ Physiotherapist/sport therapist/sport rehabilitator - da un fisioterapista/terapista dello sport/riabilitatore sportivo
- ◯ Doctor - da un medico
- ◯ Surgeon - da un chirurgo
- ◯ X-ray - tramite rx (lastra)
- ◯ MRI - tramite risonanza magnetica
- ◯ Other - altro

73. In other please describe
Se altro per favore descrivi

____

74. If your injury was diagnosed by someone, what type of injury did you sustain?
Se l'infortunio è stato diagnosticato da qualcuno, quale tipo di infortunio è stato refertato?

- ◯ bone fracture - rottura ossea
- ◯ muscle injury - lesione muscolare
- ◯ ligament tear - lesione legamento
- ◯ cartilage tear - lesione cartilaginea
- ◯ ligament + cartilage tear - lesione legamentosa + lesione cartilaginea
- ◯ other - altro

75. In other please describe

Se altro per favore descrivi

76. How the injury occur?

Come è successo l'infortunio?

- dog collision - collisione con il cane
- object collision - collisione con un oggetto
- trip - caduta
- changing direction - cambiamento di direzione
- other - altro

77. In other please describe

Se altro per favore descrivi

78. How long were you unable to do agility training for the result of your injury? (months)

Per quanto tempo sei rimasto a riposo a seguito dell'infortunio? (mesi)

79. Did you injury yourself during training or competition? (compleate one of each session for every injury)

Ti sei infortunato durante un allenamento o una competizione? (completa una di queste sezioni per ogni infortunio)

- training - allenamento
- competition - competizione

80. If training which half of th training did the injury occur?

Se durante un allenamento, durante quale metà dell'allenamento?

- first half - prima metà
- second half - seconda metà

81. If competition, which run did the injury occur?
Se durante una competizione, durante quale giro?

- 1 run
- 2 run
- 3 run
- 4 run
- any other run

82. On which kind of surface where you running?
Su quale superfice stavi correndo?

- Grass - erba
- Synthetic grass field - campo in erba sintetica
- Sand - sabbia

83. Was the injury diagnosed by someone?
L'infortunio è stato valutato/diagnosticato da qualcuno?

- Yes
- No

84. How was your injury diagnosed?
Come è stato diagnosticato il tuo infortunio?

- Physiotherapist/sport therapist/sport rehabilitator - da un fisioterapista/terapista dello sport/riabilitatore sportivo
- Doctor - da un medico
- Surgeon - da un chirurgo
- X-ray - tramite rx (lastra)
- MRI - tramite risonanza magnetica
- Other - altro

85. In other please describe
Se altro per favore descrivi

86. If your injury was diagnosed by someone, what type of injury did you sustain?
Se l'infortunio è stato diagnosticato da qualcuno, quale tipo di infortunio è stato refertato?

- bone fracture - rottura ossea
- muscle injury - lesione muscolare
- ligament tear - lesione legamento
- cartilage tear - lesione cartilaginea
- ligament + cartilage tear - lesione legamentosa + lesione cartilaginea
- other - altro

87. In other please describe
Se altro per favore descrivi

88. How the injury occur?
Come è successo l'infortunio?

- dog collision - collisione con il cane
- object collision - collisione con un oggetto
- trip - caduta
- changing direction - cambiamento di direzione
- other - altro

89. In other please describe
Se altro per favore descrivi

90. How long were you unable to do agility training for the result of your injury? (months)
Per quanto tempo sei rimasto a riposo a seguito dell'infortunio? (mesi)

91. Did you injury yourself during training or competition? (compleate one of each session for every injury)
Ti sei infortunato durante un allenamento o una competizione? (completa una di queste sezioni per ogni infortunio)

- training - allenamento
- competition - competizione

92. If training which half of th training did the injury occur?
Se durante un allenamento, durante quale metà dell'allenamento?

- first half - prima metà
- second half - seconda metà

93. If competition, which run did the injury occur?
Se durante una competizione, durante quale giro?

- 1 run
- 2 run
- 3 run
- 4 run
- any other run

94. On which kind of surface where you running?
Su quale superfice stavi correndo?

- Grass - erba
- Synthetic grass field - campo in erba sintetica
- Sand - sabbia

95. Was the injury diagnosed by someone?
L'infortunio è stato valutato/diagnosticato da qualcuno?

- Yes
- No

96. How was your injury diagnosed?
Come è stato diagnosticato il tuo infortunio?

- Physiotherapist/sport therapist/sport rehabilitator - da un fisioterapista/terapista dello sport/riabilitatore sportivo
- Doctor - da un medico
- Surgeon - da un chirurgo
- X-ray - tramite rx (lastra)
- MRI - tramite risonanza magnetica
- Other - altro

97. In other please describe

Se altro per favore descrivi

98. If your injury was diagnosed by someone, what type of injury did you sustain?

Se l'infortunio è stato diagnosticato da qualcuno, quale tipo di infortunio è stato refertato?

- bone fracture - rottura ossea
- muscle injury - lesione muscolare
- ligament tear - lesione legamento
- cartilage tear - lesione cartilaginea
- ligament + cartilage tear - lesione legamentosa + lesione cartilaginea
- other - altro

99. In other please describe

Se altro per favore descrivi

100. How the injury occur?

Come è successo l'infortunio?

- dog collision - collisione con il cane
- object collision - collisione con un oggetto
- trip - caduta
- changing direction - cambiamento di direzione
- other - altro

101. In other please describe

Se altro per favore descrivi

102. How long were you unable to do agility training for the result of your injury? (months)

Per quanto tempo sei rimasto a riposo a seguito dell'infortunio? (mesi)

103. Did you have any knee injuries in the last 5 years?
Hai avuto infortuni alle ginocchia negli ultimi 5 anni?

- ◯ yes
- ◯ no

104. How many knee injuries have you had in the last 5 years which have prevented you from doing agility? *
Quanti infortuni al ginocchio hai avuto negli ultimi 5 anni che ti hanno impedito di allenarti in agility?

	0	1	2	3	4
Left Knee	◯	◯	◯	◯	◯
Right Knee	◯	◯	◯	◯	◯

105. Thinking back to your most serious knee injury please answer the following questions
Pensando al tuo infortunio al ginocchio peggiore, rispondi alle seguenti domande

106. On which kind of surface where you running?
Su quale superfice stavi correndo?

- ◯ Grass - erba
- ◯ Synthetic grass field - campo in erba sintetica
- ◯ Sand - sabbia

107. Was the injury diagnosed by someone?
L'infortunio è stato valutato/diagnosticato da qualcuno?

- ◯ Yes
- ◯ No

108. How was your injury diagnosed?
Come è stato diagnosticato il tuo infortunio?

- ◯ Physiotherapist/sport therapist/sport rehabilitator - da un fisioterapista/terapista dello sport/riabilitatore sportivo
- ◯ Doctor - da un medico
- ◯ Surgeon - da un chirurgo
- ◯ X-ray - tramite rx (lastra)
- ◯ MRI - tramite risonanza magnetica
- ◯ Other - altro

109. In other please describe
Se altro per favore descrivi

110. If your injury was diagnosed by someone, what type of injury did you sustain?
Se l'infortunio è stato diagnosticato da qualcuno, quale tipo di infortunio è stato refertato?

- ◯ bone fracture - rottura ossea
- ◯ muscle injury - lesione muscolare
- ◯ ligament tear - lesione legamento
- ◯ cartilage tear - lesione cartilaginea
- ◯ ligament + cartilage tear - lesione legamentosa + lesione cartilaginea
- ◯ other - altro

111. In other please describe
Se altro per favore descrivi

112. How the injury occur?
Come è successo l'infortunio?

- ◯ dog collision - collisione con il cane
- ◯ object collision - collisione con un oggetto
- ◯ trip - caduta
- ◯ changing direction - cambiamento di direzione
- ◯ other - altro

113. In other please describe

Se altro per favore descrivi

114. How long were you unable to do agility training for the result of your injury? (months)

Per quanto tempo sei rimasto a riposo a seguito dell'infortunio? (mesi)

115. Have you changed your training practices as a result of your knee injury (e.g. alter lenght intensity of sessions, avoidance of certain activity) if yes please describe.

Hai cambiato le tue abitudini di allenamento a causa del tuo infortunio al ginocchio? (es lunghezza ed intensità delle sessione, evitare certe attività) se si descrivi

116. Have you ever had any injection to either of your knees? if yes please specify what type of injection and to which knee

Hai subito infiltrazioni a uno o entrambe le ginocchia? se si specifica quale tipo di infiltrazione e su quale ginocchio

117. Have you ever had any surgery to either of your knees? if yes specify what type of surgery and to witch knee

Hai avuto interventi chirurgici ad uno o entrambe le ginocchia? se si specigica quale tipo di intervento su quale ginocchio

"Non agility" training / Sports

118. How many session of "non agility training" do you usually participate in each week?

Quante sessioni di allenamento diverso dall'agility pratichi a settimana?

- ◯ Zero
- ◯ 1
- ◯ 2
- ◯ 3
- ◯ More

119. How long does each non agility training session usually last? (in hours)

Quanto dura di solito ogni sessione di allenamento diverso dall'agility? (in ore)

120. What type of non agility training do you usually do in the non agility training sessions?

Quale tipo di allenamento diverso dall'agility pratichi durante le sessioni?

- ☐ Weight trainig - reisitance machines / Allenamento con i pesi - utilizzando macchine
- ☐ Weight training- free weight (e.g. dumb bells, bar bells) / allenamento con i pesi - utilizzando pesi
- ☐ Plyometric training - jumping/hopping/bounding / allenamento pliometrico - slati/balzi/movimenti esplosivi
- ☐ Balance training
- ☐ Class/group-based training - weight bearing (circuit training, body pump) / Allenamenti di gruppo - con i pesi (allenamenti a circuito, body pump)
- ☐ Class/group-based training - non weight bearing (e.g. spinning) / allenamenti di gruppo senza pesi (spinning)
- ☐ Body weight training - callisthenics (e.g press up, pull ups) / allenamenti che utilizzano il peso corporeo - callistenics (flessioni, trazioni)
- ☐ Yoga
- ☐ Cross-fit
- ☐ Distance running - corsa
- ☐ Distance cycling - ciclismo
- ☐ Dog obedience
- ☐ Disc dog
- ☐ Canicross, Bike jouring
- ☐ Other

121. What is your main reason for non agility training? (plase check all the apply)

Qual'è lo scopo principale per cui pratichi un'allenamento diverso dall'agility? (seleziona tutte le vere)

- [ ] Improve performance - migliorare le performance
- [ ] Improve skills - migliorare la tecnica
- [ ] Lose weight - perdere peso
- [ ] Improve muscolar tone - migliorare il tono muscolare
- [ ] Fitness
- [ ] Socialising - socializzare
- [ ] Activity with your dog - fare attività col cane
- [ ] Socializing your dog - socializzare il tuo cane

122. Answer only in you don't do any weight training, if you do skip this question.
If you do not engage in any kind of weight trining, please outline your main reason why:

Rispondi solo se non pratichi sollevamento pesi, se lo fai passa oltre questa domanda.
Se non pratichi allenamento con i pesi, per favore specifica la principale ragione:

- [ ] would make me too bulky - tempo di diventare troppo muscoloso/a
- [ ] lack of confidence in lifting weights - poca sicurezza nel sollevare pesi
- [ ] Don't think i need to do it - non penso di averne bisogno
- [ ] Don't have time - non ho tempo
- [ ] I don't know the best excercises or how to do them - non saprei quali esercizi fare o come farli
- [ ] Lack of access to equipment and facilities - Mancanza di accesso a strutture e materiali
- [ ] Other - altro

123. Answer this question only if you don't do any non agility session or you do only weight training excercize. If you do skip this question
Other then weight training, if you do not engage in any other king of non-agility training, please outline main reason why:

Rispondi a questa domanda solo se non fai nessuna sessione di allenamento extra agility o le tue sessioni comprendono solo esercizi con i pesi. Se fai altri tipi di allenamento salta questa domanda.
Se non fai nessuna sessione di allenamento extra agility che non comprendano esercizi con i pesi, spiega perchè:

- [ ] Lack of confidence in exercise - poca sicurezza nel eseguire gli esercizi
- [ ] Don't think i need to do it - non penso di averne bisogno
- [ ] Don't have time - non ho tempo
- [ ] I don't know the best excercises or how to do them - non saprei quali esercizi fare o come eseguirli
- [ ] Can't afford to pay for facilities - non posso permettermi di pagare la palestra

124. On a scale of 1-4 how beneficial do you belive the following activities are for a agility athlete? (tick one box only for each activity);

In una scala tra 1 e 4 quanto pensi che queste attività possano beneficiale al conduttore di agility? (seleziona un box per ogni attività)

	1 Not beneficial - Non beneficia	2 Slightly beneficial - Beneficia poco	3 Beneficial - Può portare poco beneficio	4 Highly beneficial - Porta molto beneficio
Weight trainig - reisitance machines / Allenamento con i pesi - utilizzando macchine	○	○	○	○
Weight training- free weight / allenamento con i pesi - utilizzando pesi	○	○	○	○
Plyometric training - jumping/hopping/bounding / allenamento pliometrico - slati/balzi/movimenti esplosivi	○	○	○	○
Balance training	○	○	○	○
Class/group-based training - weight bearing / Allenamenti di gruppo - con i pesi (allenamenti a circuito, body pump)	○	○	○	○
Class/group-based training - non weight bearing / allenamenti di gruppo senza pesi (spinning)	○	○	○	○
Body.weight training - callisthenics / allenamenti che utilizzano il peso corporeo - callistenics (flessioni, trazioni)	○	○	○	○
Yoga	○	○	○	○
Cross-fit	○	○	○	○
Distance running / corsa	○	○	○	○
Distance cycling / ciclismo	○	○	○	○
Dog obedience	○	○	○	○
Disc dog	○	○	○	○
Canicross, Bike juring	○	○	○	○

Other	◯	◯	◯	◯

Microsoft Forms
